# Potentially avoidable inpatient nights among warfarin receiving patients; an audit of a single university teaching hospital

**DOI:** 10.1186/1756-0500-2-41

**Published:** 2009-03-13

**Authors:** Dónall Forde, Mortimer B O'Connor, Oonagh Gilligan

**Affiliations:** 1 The School of Medicine, University College Cork, Cork, Ireland; 2 The Department of Medicine, Cork University Hospital, Cork, Ireland; 3 The Department of Haematology, Cork University Hospital, Cork, Ireland

## Abstract

**Background:**

Warfarin is an oral anticoagulant (OAT) that needs active management to ensure therapeutic range. Initial management is often carried out as an inpatient, though not requiring inpatient facilities. This mismatch results in financial costs which could be directed more efficaciously. The extent of this has previously been unknown. Here we aim to calculate the potential number of bed nights which may be saved among those being dose optimized as inpatients and examine associated factors.

**Methods:**

A 6 week prospective audit of inpatients receiving OAT, at Cork University Hospital, was carried out. The study period was from 11^th ^June 2007 to 20^th ^July 2007. Data was collected from patient's medications prescription charts, medical record files, and computerised haematology laboratory records. The indications for OAT, the patient laboratory coagulation results and therapeutic intervals along with patient demographics were analysed. The level of potentially avoidable inpatient nights in those receiving OAT in hospital was calculated and the potential cost savings quantified. Potential avoidable bed nights were defined as patients remaining in hospital for the purpose of optimizing OAT dosage, while receiving subtherapeutic or therapeutic OAT (being titred up to therapeutic levels) and co-administered covering low molecular weight heparin, and requiring no other active care. The average cost of €638 was taken as the per night hospital stay cost for a non-Intensive Care bed. Ethical approval was granted from the Ethical Committee of the Cork Teaching Hospitals, Cork, Ireland.

**Results:**

A total of 158 patients were included in the audit. There was 94 men (59.4%) and 64 women (40.6%). The mean age was 67.8 years, with a median age of 70 years.

Atrial Fibrillation (43%, n = 70), followed by aortic valve replacement (15%, n = 23) and pulmonary emboli (11%, n = 18) were the commonest reasons for prescribing OAT. 54% had previously been prescribed OAT prior to current admission.

It was confirmed that, there are potentially avoidable nights in patients receiving OAT. The majority of this group were those being commenced on OAT for the first time (p = 0.00002), in the specialities of Cardiology, Cardiothoracic surgery and Care of the Elderly. The potential number of bed nights to be saved is 13 per week for the hospital or 1.1 bed nights per 10,000 general hospital admissions. These were predominantly weekday nights. The estimated cost of avoidable inpatient OAT dose optimization was approximately €8300 per week.

**Conclusion:**

With rising costs and the increasing demands for acute hospital beds, alterations to inpatient management for this group of patients should be considered. Alternatives include increasing the size of current anticoagulation clinics, introduction of POCT (point of care testing) devices and increased GP management. POCT can be justified based upon the publication by *Gardiner et al*, who showed that 87% of patients find self testing straightforward, 87% were confident in the result they obtained using the devices and 77% preferred self testing.

## Background

Warfarin, a Vitamin K antagonist, is the most commonly prescribed oral anticoagulant therapy (OAT). Patients taking this OAT must have regular coagulation measurements to assess appropriate OAT dosage. Subtherapeutic anticoagulation can result in thrombosis, which can be life-threatening, while supratherapeutic anticoagulation can result in haemorrhage, which can also be fatal. The commonest indications for OAT are prevention of arterial thromboembolism in patients with atrial fibrillation and/or mechanical heart valves and treatment and prevention of deep vein thrombosis and pulmonary emboli. Duration of treatment varies, from 6 weeks to 6 months in venous thrombosis, to lifelong therapy in cardiac indications or recurrent thrombosis. The risk of haemorrhage on long-term OAT varies between 1 and 15% per annum and the risk of death increases with increasing coagulation, accepting that death may be due to co-morbidity, an elevated coagulation status being an indicator of end stage disease [1].

It is the opinion of the authors that patients are often needlessly kept in hospital as inpatients solely for the purpose of optimizing OAT to achieve adequate anticoagulation. This service does not require inpatient facilities and thus a mismatch of services to requirement is occurring, with financial consequences. Alternatives to this inpatient management are to increase the facilities of the hospitals current anticoagulation clinics, introduction of point of care testing (POCT) devices and increase General Practitioner management of OAT. This study aims to confirm and quantify patients remaining as inpatients solely for the purpose of optimizing OAT dosage.

## Methods

A 6 week prospective audit of all inpatients receiving OAT, at Cork University Hospital, a hospital with approximately 27,000 annual inpatient admissions, was carried out. The study period was from 11^th ^June 2007 to 20^th ^July 2007. The study period was selected to include 3 weeks pre and post doctor change over. Note 1^st ^July is the changeover date for new non-consultant hospital doctors.

Data was collected from patient's medications prescription charts, medical record files, and computerised haematology laboratory records. The indications for OAT, patient laboratory coagulation results and therapeutic intervals along with patient demographics were recorded by a single data recorder researcher and analysed. On patient discharge, contact was made with the primary admission team and patient records were reviewed by the single data recorder researcher, to establish if the patient had spent potentially avoidable nights, solely due to OAT management. Judgement regarding potentially avoidable nights was made by the single data recorder researcher on review of patient medical files. Pre-specified criteria for a potentially avoidable bed night was defined as a patient remaining in hospital for the purpose of optimizing OAT dosage, while receiving subtherapeutic (being titred up to therapeutic levels) or therapeutic OAT and co-administered covering low molecular weight heparin, and requiring no other active care. Primary admission teams' opinions were also recorded.

The level of potentially avoidable inpatient nights in those receiving OAT was calculated and the potential cost savings quantified. The average cost of €638 was taken as the per night hospital stay cost for a non-Intensive Care Unit bed in our hospital. Data analysis was carried out using SPSS.

Ethical approval was granted from the hospital's Quality of Research Department and the Ethical Committee of the Cork Teaching Hospitals, Cork, Ireland. Informed consent was received from participants.

## Results

A total of 158 patients received OAT as an inpatient during the study period. There were 94 men (59.4%) and 64 women (40.6%), with a mean age of 67.8 years (median = 70). 54% (n = 86) were prescribed OAT prior to study period admission, of which 68% (n = 59) had a therapeutic anticoagulation status on admission.

The commonest reasons for prescribing OAT, among the cohort, was Atrial fibrillation (43%, n = 70), followed by atrial valve replacement (15%, n = 23), pulmonary embolus (11%, n = 18), Deep vein thrombosis (8%, n = 12) and cerebrovascular accidents (3%, n = 4). Therefore, understandably, the specialties of Cardiothoracic Surgery, Cardiology and Care of the Elderly were the biggest prescribers of OAT, comprising 46% (73/158) of cases (20%, n = 31; 17%, n = 27; 9%, n = 15 respectively).

On review of patient's medications prescription charts, medical record files, and computerised haematology laboratory records, a total of 26 patients (26/158 = 16.5%) were noted to have at least one potentially avoidable night in hospital due to OAT dosage optimization alone. The potentially avoidable bed nights for these patients totals 78 beds in the 6 weeks, with a mean of 3 potentially avoidable nights per patient (median = 2, mode = 2). Of interest there was a statistically significant relationship between whether the patient was newly prescribed OAT (n = 72) or previously had OAT prescribed prior to study admission (n = 86), and whether they spend potentially avoidable nights in hospital. 86.7% of patients treated with OAT prior to admission spent no potentially avoidable nights in hospital whereas 52.9% of the new OAT patients spent a potentially avoidable night in hospital (p-value = 0.00004). Of those patients who spent potentially avoidable nights in hospital for OAT dosage optimization, 58% (15/26) spent at least one weekend night as an inpatient, i.e. a Saturday and/or Sunday night. The majority of avoidable nights were during the week, 73% (57/78). Of the 86 existing OAT prescribed patients, 36% were therapeutic or achieved optimization of their OAT (up 10% upon their admission) by discharge, while of the 72 new patients discharged 34% were therapeutic. The median, mode and mean number of coagulation blood test measurements taken in hospital for the cohort were 8, 9 and 9.62 respectively.

The mean stay of the cohort patients was 12.9 days (median = 10, mode = 9), with the mean stay of patients who spent a potentially avoidable night of 13.7 days (median = 10.5, mode = 6).

Regards a doctor/team perspective on avoidable nights, admitting team doctors reported that in 16.5% (26/158) of cases they felt that there was at least one potentially avoidable night in hospital as a consequence of inpatient OAT dosage optimization. These 26 cases highlighted by treating doctors coincided with all of the 26 cases found by the single data recorder researcher. Variations were seen in pre and post the 1^st ^July (changeover date for new non-consultant hospital doctors). Pre 1^st ^July, 21% (n = 15) of cases had at least one potentially avoidable night in hospital for the purpose of OAT dosage optimization, compared to 12.5% (n = 11) in the three weeks post 1^st ^July changeover.

## Discussion

From the findings of this study one may draw the conclusion that there are potentially avoidable nights in those initiated on OAT in hospital. In this study, avoidable hospital nights occurred in 26 out of 158 patients. These nights totalled 13 per week or 676 a year. The average care cost per non-ICU bed for the hospital is €638, giving cost of €8300 per week, and therefore these beds are worth managing more efficaciously.

Of the 26 patients who spent potentially avoidable nights in hospital, 15 had at least one night occurring at the weekend. Although the majority of the potentially avoidable nights did occur during the week, some are occurring at the weekend. This is a time when no active elective medical management is taking place, re-enforcing the point that these nights are avoidable both for patient and hospital.

By looking at the other variables, it was found that it is a specific sub-population of OAT receiving patients that make up the majority of those 26 patients. 18 out of the 26 patients were newly prescribed OAT on their current admission. Significantly more potentially avoidable nights were required for patients new to OAT (p = 0.00004). When taken in addition to the result that the 3 biggest contributing departments (Cardiothoracic Surgery, Cardiology and Care of the Elderly) comprise 46% of the study cohort, the majority of the OAT prescribed population in question can be identified. This makes targeting changes easier.

Taking the average number of inpatients nights in the total population versus those that could have gone home earlier there is a small difference of 2.4 days (12.9 days vs.10.5 days). The mean number of potentially avoidable nights is 3 per patient. So an average stay of 10.5 days minus average potentially avoidably stay of 3 days equals almost a third of these patients inpatients duration may be avoidable.

Of the 86 patients who had previous experience of OAT, 26% were therapeutic on admission, while on their discharge 36% were therapeutic (up 10% upon their admission). It can be seen that of the 72 new patients discharged only 34% were therapeutic. Although no correlation between therapeutic on admission/discharge with potentially avoidable nights was found, it gives a baseline to which the efficacy of OAT is being managed, and thus allowing re-audits in the future a point of reference. Out of a mean 10 coagulation blood test measurements taken in hospital per patient 7 were sub-therapeutic.

For the management of OAT receiving patients other options include the expansion of the anticoagulation clinics and involving General Practitioners in outpatient care. This must be carried out within a means which would be financially appropriate. One issue to this may be the quality of the continuity of care. If these patients are to be managed effectively in an outpatient manner then the requests of the hospital based teams need to be, both clearly documented and freely available to our out-patient colleagues. The value of this has been shown by an American study which showed that patients with a work up error were 6.2 times more likely to be re-hospitalised within 3 months [2]. This work-up error is when an out-patient test or procedure suggested or scheduled by the inpatient provider was not adequately provided by the outpatient provider [2] i.e. a coagulation blood test check and resultant change of OAT dose. This need for better coordination and a systematic approach can also be reinforced from the findings of the study by van Walraven C and colleagues which found that patients from community practices showed significantly worse anticoagulation control than those from anticoagulation clinics [3]. The Anticoagulation Forum in the United States also feels that a systematic approach is the key to anticoagulation management [4].

Another option, to avoid inpatient OAT dosage optimization, is further devolution of management to GPs. The acceptance of such responsibility is dependant on those practitioners being knowledgeable and confident that their practice has the abilities to deliver such a service, along with being aware of hospital requests and formulated patient specific protocols. In a study of Norwegian GPs, gross variations in practice were noted along with an unrealistically high risk of severe bleeding when faced with the management of a moderately high INR anticoagulation measurements of 5.9 [5]. This could explain our primary care givers reluctance to take over management of warfarin patients [6]. Computer dose calculation programs are available and this along with training in OAT management will lead to an effective outlet to save on costs due to avoidable inpatient stays.

The final option is POCT devices. These devices are attractive due to their superior efficacy in terms of time spent therapeutic versus routine care, as well as lower incidence of adverse events recorded [7-9]. Not every patient is suitable but, in who are, mainly long term OAT patients who remain stable, it is a welcome therapeutic option [7]. Out of every 100 eligible patients approximately 14 would be able to conduct long-term self monitoring [8]. A locally carried out randomised controlled trial, which remains unpublished, took 163 patients from the same hospital and combined anticoagulation management service AMS with the POCT devices. The patients self-tested and logged onto the internet and were advised of dose. If therapeutic they were automatically provided algorithm-derived dosing and repeat testing instructions. Those with non-therapeutic results were prioritized for pharmacist review. Gardiner et al suggest that 87% of patients find self testing straightforward, 87% were confident in the result they obtained using the devices and 77% preferred self testing [10].

From a financial perspective Boucher M and colleagues have shown that converting from inpatient to outpatient treatment of the condition of a proximal DVT was associated with a significant cost savings for their Canadian institution, with no apparent compromise in patient care noted [11].

Despite all of the above leaning towards the complete initiation and management of OAT in the OPD setting, in certain circumstances inpatient initiation of OAT is recommend in the inpatient setting. The reason for such is when there are poor links between community and hospital systems and the risk of adverse events to OAT are high, such as in patients with a history of alcohol abuse, chronic renal insufficiency, and a previous gastrointestinal bleed [12]. Each case should be dealt with individually, but if good links between inpatient and outpatient services exist and there is minimal adverse event risk outpatient care of OAT should be strongly considered.

## Conclusion

Despite patients being kept in hospital, for many nights, solely for OAT dosage optimization, many are still discharged with subtherapeutic levels.

With rising costs and the increasing demands for acute hospital beds, alterations to inpatient management for this group of patients should be considered. Alternatives include increasing the size of current anticoagulation clinics, introduction of POCT devices and increased GP management. POCT can be justified based upon Gardiner et al, who showed that 87% of patients find self testing straightforward, 87% were confident in the result they obtained using the devices and 77% preferred self testing.

In our hospital there is a potential to save approximately 1.1 bed nights per 10,000 general hospital admissions or €8300 per week, should an alternative to inpatient OAT dosage optimization be achieved.

Future work including a re-audit after implementation of recommended changes would be advisable. As this is a single centre study the authors would recommend a multicenter study over a longer study period to verify results. It is hoped this study will act as a starting point for more in-depth research in this area of medicine.

## Competing interests

The authors declare that they have no competing interests.

## Authors' contributions

DF prepared the study design, carried out data collection, statistical analysis, prepared the manuscript. MOC prepared the manuscript. OG prepared the study design.

All authors read and approved the final manuscript.
